# Clinical diagnostic reference levels and image quality metrics for CT in oncology patients

**DOI:** 10.4102/sajr.v28i1.2960

**Published:** 2024-10-31

**Authors:** Ida-Keshia Sebelego, Sussan Acho, William I.D. Rae

**Affiliations:** 1 Department of Clinical Sciences, Faculty of Health and Environmental Sciences, Central University of Technology, Bloemfontein, South Africa; 2 Department of Medical Physics, Faculty of Health Sciences, University of the Free State, Bloemfontein, South Africa; 3 Department of Medical Imaging, Prince of Wales Hospital, Randwick, Australia

**Keywords:** cancer, computed tomography, local diagnostic reference level, image quality index, size-specific dose estimates

## Abstract

**Background:**

In CT, the volumetric CT dose index (CTDI_vol_), dose-length product (DLP) and patient’s size-specific dose estimates (SSDE) are used as diagnostic reference level (DRL) metrics.

**Objectives:**

To develop clinical local DRL values for CT chest-abdomen-pelvis (CAP) examinations using the CTDI_vol_, DLP and SSDE, and to determine the image quality achieved.

**Method:**

In total, 201 cancer patients were included in the study. The scanning parameters, dose metrics from the CT unit and participants’ body mass index (BMI) were documented. The local CT DRL values for CAP examinations were defined as the median and 75th percentiles of the dose distribution.

**Results:**

The local DRL values given in terms of median CTDI_vol_ ranged between 8.4 mGy and 12.7 mGy for the different types of cancers. The median DLPs ranged from 848 to 1173.4 mGy.cm for the various cancers. Generally, the radiation dose was directly proportional to the BMI and number of scan phases. Significant differences were observed between the DRLs for the various size-related parameters, number of scan phases and BMI classifications. The image quality was clinically satisfactory.

**Conclusion:**

No baseline data for clinical DRL values were available for this medical oncology department. The achieved DRLs were similar to published size-specific DRLs. The image quality was maintained during CT imaging. Dose optimisation and image quality assessment should be implemented to ensure optimal scanning parameters for different cancers in CT CAP examinations.

**Contribution:**

The first size-specific local DRL values for CT CAP investigations carried out on oncology patients in South Africa have been established.

## Introduction

Justification and optimisation are two important principles when ionising radiation is used for medical purposes.^1^ The International Commission on Radiological Protection (ICRP) emphasises optimisation, in particular, relating to all exposure situations (planned, emergency and existing). Radiology departments must therefore evaluate how this principle is applied in their respective departments when carrying out examinations requiring ionising radiation. Optimisation involves applying the ‘as low as reasonably achievable’ (ALARA) principle, which does not involve initiating dose limits while providing diagnostic image quality.^1^

In planned diagnostic medical imaging such as computed tomography (CT), calculating the diagnostic reference level (DRL) values contributes to the optimisation of patient doses delivered for this imaging modality.^1^ Various authors have established DRL values for CT using either the volumetric CT dose index (CTDI_vol_) and/or dose-length product (DLP) metrics.^2,3,4,5,6,7,8^ Size-specific dose estimates (SSDE), based on the patient’s size, are another metric to define DRL values, introduced by the American Association of Physicists in Medicine (AAPM)^9^. Although several studies have investigated the application of SSDE for CT examinations,^10,11,12,13^ the SSDE is not widely used as it is not routinely generated on the patient radiation dose structured report produced by CT units, contrary to the CTDI_vol_ and DLP.^14,15^

Clinical DRL values have also been published based on the CTDI_vol_ and DLP,^16,17,18^ and only two studies have been published presenting clinical DRL values (CTDI_vol_ and DLP) that were patient size specific.^17,19^ No oncology-specific clinical DRL values for SSDE based on the size of the patient could be traced in the literature.

The clinical indication protocol further influences the number of scan phases and in cases of multiphase examinations, the dose to the patient. Some multiphase CT examinations can consist of two to four scan phases depending on the clinical indication and protocol. These phases may include the non-contrast phase, arterial phase, portal venous or venous phase and delayed or late phase. During the arterial phase, obtained at 35 s – 40 s after contrast medium injection, pathologies such as hepatocellular carcinoma and adenoma in the liver can be identified.^20^ The portal venous phase, obtained at 70 s – 80 s after the injection of contrast medium, can assist in enhancing hypo-vascular liver lesions.^20^ At the participating medical oncology department in this study, a 70 s delay is used for the portal venous phase. The delayed or late phase is performed 3 min – 10 min after the injection of contrast medium to assess aspects such as leakage of contrast medium,^21^ excretion by the kidneys^22^ and to detect pathologies that were not clear on a portal venous phase, such as renal cell carcinoma,^22^ renal and adrenal masses.^21^ At the participating hospital, two scans are normally obtained for CT chest-abdomen-pelvis (CAP) examinations, namely an arterial together with the portal venous phase. As the number of scan phases increases for a CT examination, the dose increases.^23,24^ Therefore, when DRL values are calculated, multiphase examinations should be calculated separately from single-phase CT examinations.^25^

The South African Health Products Regulatory Authority (SAHPRA) also recommends DRL values for the most commonly performed examinations in medical imaging.^26^ Diagnostic reference level values for CT in South Africa have been compiled based on the anatomical region^27^ and the first South African clinically indicated size-specific CT DRL values have been reported.^28^ To the authors’ knowledge, no oncology-specific clinical DRL values based on the patient’s size and the SSDE have been published. Consequently, the aim of this study was to compile size-related patient doses based on the clinical indication for cancer and different types of cancer for various image quality metrics.

## Research methods and design

Two hundred and one patients aged 18 years or older, who were scheduled for CT CAP examinations and provided informed consent to have their weight and height measured for calculation of their body mass index (BMI), were included in the study. The clinical indication for all the patients was cancer-related. The CT CAP examinations that formed part of the study were for diagnostic purposes and not for radiation therapy planning. The participants were not traced for other dose-related examinations or treatments apart from the CT CAP examinations for cancer evaluation, oncologic follow-up and/or staging. The different types of cancer were grouped according to anatomical systems. Cervix, uterus, vulva, ovarian and endometrial cancer were grouped together as part of the female reproductive system. Oesophageal, stomach, colon or rectosigmoid, anal, rectal and caecal cancer formed part of the gastrointestinal tract (GIT) of the digestive system. Breast cancer was the most common clinical indication for the CT CAP examination in this study and was not grouped with another anatomical system.

Before the commencement of data collection, the MDW 300 L scale (Adam Equipment SA [Pty] Ltd; Johannesburg, South Africa), used to measure the BMI, was calibrated. Patient data were collected from 01 March 2021 to 10 June 2021. The medical oncology department and the patients for the study were selected using a convenience sampling technique. The sample (201 patients) was divided into four different groups according to their BMI classifications (underweight: < 18.5 kg/m^2^; normal weight: 18.5 kg/m^2^ – 24.9 kg/m^2^; overweight: 25 kg/m^2^ – 29.9 kg/m^2^; and obese: ≥ 30 kg/m^2^) using stratified random sampling (SRS) to calculate the patient dose according to their BMI classification.

Data were obtained from the GE BrightSpeed 8 CT unit (General Electric Healthcare; Chicago, IL, USA) that was installed in 2007 at the medical oncology department as a demonstration unit. The scan was performed with a body filter, using a standard convolution kernel in helical acquisition mode. The department used an image reconstruction algorithm and automatic tube current modulation (ATCM). The ATCM used was SmartmA and AutomA, while the reconstruction algorithm used was the filtered back projection (FBP). One protocol was used to scan all 201 patients scheduled for CT CAP examinations. The CT acquisition parameters are displayed in Table 1. At the start of data collection, the pitch was 1.675, but the pitch was later changed to 1.35 for the protocol. Only the number of scan phases, the scan length and scan time varied among the patients. The CT images included in the study were reported by radiologists at the medical oncology department.

**TABLE 1:** Computed tomography imaging acquisition parameters showing the mean (minimum–maximum range) based on the CT chest-abdomen-pelvis cancer examinations.

Feature	Protocol parameters
Kilovoltage (kV)	120
Milliampere (mA)	440
Pitch	1.675; 1.35
Scan length (mm)	349 (245–495)
Scan time (s)	6.9 (5.0–9.5)
Rotation time (s)	0.50
ATCM image quality indicator (NI)	11.5

ATCM, automatic tube current modulation; CT, computed tomography; NI, noise index.

### Research instrument

A patient-dose document that was quantitative in nature was compiled by the researcher and benchmarked with the literature^29,30^ for use in this study. The patient-dose document descriptively collected specific information from the CT unit and measurements from the patients that were used to develop DRL values. This included information pertaining to the patient such as the BMI, weight and age (obtained prior to the examination after informed consent had been provided). Numerical data such as the rotation time, pitch, CTDI_vol_ and tube current from the CT unit were captured on the patient-dose document for each patient in the study. Information such as the clinical indication and use of contrast medium was recorded.

All the CT images were stored on an external hard drive in Digital Imaging and Communications in Medicine (DICOM) format so that anterior-posterior (AP) and lateral (LAT) measurements on the CT images could be determined, measured using electronic callipers on a MicroDicom (mDicom) viewer (MicroDicom Ltd.; Sofia, Bulgaria),^28^ in order to calculate the effective diameter, and recorded on the patient-dose document. No patient names were mentioned, and each patient received a unique number. All the data obtained for the study were password-protected. The patient-dose documents and the external hard drive were kept in a secure cupboard that could only be accessed by the principal researcher.

### Dose metric – Size-specific dose estimates

The five patient size-related parameters are the sum of AP and LAT dimensions, AP dimension, effective diameter, LAT dimension and BMI (non-specific) for SSDE calculation.^28^ The parameter BMI (non-specific) refers to grouping all BMI classifications together. Equation 1, derived from the AAPM,^9^ was used to calculate the SSDE for the size-related parameters, while the size-related parameter, BMI, was calculated using the conversion factors published by O’Neil et al.^31^ The SSDE_BMI (non-specific)_ refers to the SSDE that was calculated using the patient’s BMI. Thus, the patient size (BMI) was taken into consideration to calculate the SSDE:

$$\text{SSDE}={\text{CTDI}}_{\text{vol}}\text{ X conversion factor}$$ [Eqn 1]

### Data analysis

The data from the patient-dose document were transferred to a Microsoft Excel Version 365 (Microsoft Corporation; Redmond, WA, USA) spreadsheet and minimum, maximum and mean values were determined. The medians (50th percentile) and 75th percentiles were established for the CTDI_vol_, DLP and the five size-related parameters for SSDE^28^ for CT CAP examinations with cancer as the clinical indication. The Kruskal–Wallis test was used to compare median values to establish any significant differences among the DRL quantities or metrics, BMI classifications, specific cancers and the number of scan phases. Furthermore, the Wilcoxon two-sample test was used to compare the size-related parameters for SSDE between overweight and obese patients. To determine any significant differences between size-related parameters, a cut-off value of *p* < 0.05 was used. Furthermore, the Spearman correlation coefficient was used to investigate whether a correlation between the size-related parameters and DRL quantities was noted. A significant correlation between the size-related parameters and DRL quantities was assumed when *p* < 0.05.

### Image quality index

**FIGURE 1:**
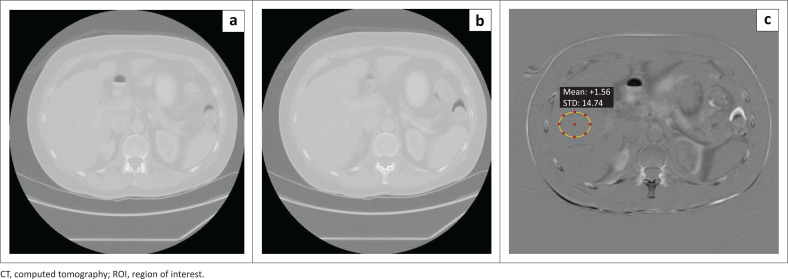
An example of the subtraction process for image quality analysis. (a) and (b) are adjacent images, and therefore independent images in the CT dataset. The difference between (a) and (b) is given as (c). On the subtracted image (c), an elliptical ROI is placed on the liver providing the mean and standard deviation in Hounsfield units.

For each patient CT image sequence, adjacent images were subtracted from each other using the MATrix LABoratory (MATLAB) R2017a (The Math Works, Inc., Natick, MA, USA).^28^ The region of interest (ROI) was placed on the liver of the subtracted images for eight to 10 sequential slices per patient, as illustrated in Figure 1. In a study by Moghadam et al.,^11^ the ROI was only drawn on the liver for an abdomen-pelvis examination. The mean standard deviation of the Hounsfield unit (HU) values of the ROI of these slices was calculated. It was assumed the structured noise because of small anatomical structures within the liver contributed a constant offset to the quality index. The image quality index was expressed as the standard deviation divided by root two to account for the subtraction process.^32^

### Ethical considerations

The Health Sciences Research Ethics Committee (HSREC) of the University of the Free State in Bloemfontein, South Africa (Ethics clearance number: UFS-HSD2019/0612/011002) provided ethical approval for the research. Permission was granted by the Free State Province Department of Health to acquire patient information from the CT unit because data were collected at a public health institution. Written informed consent was provided by all the patients who participated in the study.

## Results

A total of 201 adult patients were referred to the medical oncology department for CT CAP examinations during the period 01 March 2021 to 10 June 2021. Three-quarters (*n* = 151; 75.0%) of the patients were females. The patients’ age ranged between 22 years and 87 years (median age 52 years). All the patients referred for a CT CAP examination had cancer-related clinical indications such as breast, penile, lung, rectal and vulva cancer. One-third (*n* = 65; 32.3%) of the patients displayed a normal BMI (18.5 kg/m^2^ – 24.9 kg/m^2^). Only 16 (8.0%) patients were underweight. The remaining 120 (59.7%) patients were either overweight or obese.

**FIGURE 2:**
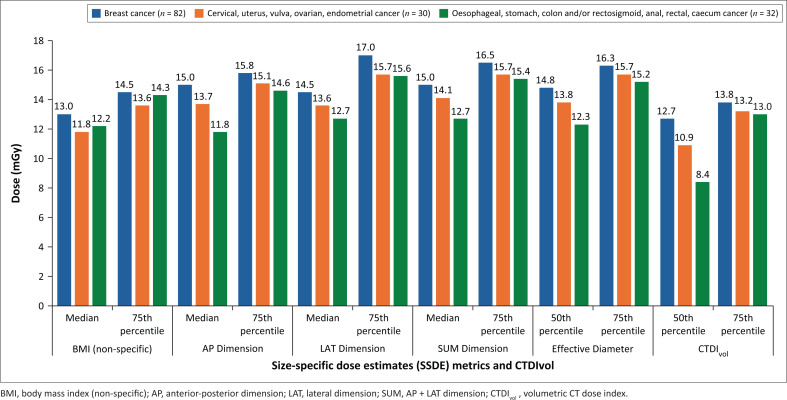
Clinical diagnostic reference level values (median and 75th percentile) in terms of size-specific dose metrics for (1) breast cancer, (2) cervical, uterus, vulva, ovarian and endometrial cancer and (3) oesophageal, stomach, colon and/or rectosigmoid, anal, rectal and caecum cancer who had 20 or more patients per category.

**FIGURE 3:**
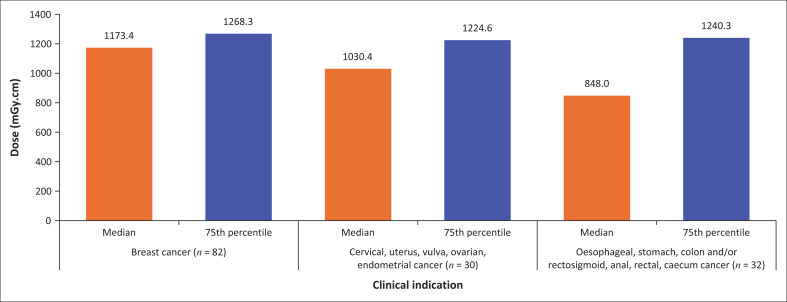
Clinical diagnostic reference level values (median and 75th percentile) for the dose-length product for (1) breast cancer, (2) cervical, uterus, vulva, ovarian and endometrial cancer and (3) oesophageal, stomach, colon and/or rectosigmoid, anal, rectal and caecum cancer who had 20 or more patients per category.

The findings in this study are the first set of clinical local DRL (LDRL) values (median and 75th percentile) at this particular medical oncology department for CT CAP clinical size-specific DRL values for specific types of cancer. The three main cancers recorded in this study were, (1) breast cancer (two scan phases) with 82 patients, (2) cervical, uterus, vulva, ovarian and endometrial cancer (two scan phases) with 30 patients and (3) oesophageal, stomach, colon and/or rectosigmoid, anal, rectal and caecum cancer (two scan phases) with 32 patients (Figure 2 and Figure 3).

For penile, testicular and prostate cancer (male reproductive system), only 13 patients underwent two scan phases, and one patient underwent three scan phases. In relation to renal and bladder cancer (urinary system), only three patients underwent two scan phases. The same trend of less than 20 cases was noted for (1) pancreas, liver and bile duct cancer (two scan phases), (2) breast cancer (three scan phases), (3) cervical, uterus, vulva, ovarian and endometrial cancer (three scan phases), (4) oesophageal, stomach, colon and/or rectosigmoid, anal, rectal and caecum cancer (three scan phases), (5) sarcoma, scalp squamous cell carcinoma and melanoma (two scan phases), (6) lung cancer (two scan phases) and (7) the exclusion or query of cancer (two scan phases).

Figure 2 and Figure 3 illustrate the clinical LDRL values for specific cancers based on various dose metrics. Table 2 gives the summary of the clinical LDRL values for non-specific cancers and a number of scan phases as the median and 75th percentile for the different dose metrics based on the non-specific BMI and BMI classifications. Significant differences ranging from < 0.0001 to 0.0321 were noted between the use of two and three scan phases based on the radiation dose the patient received. A strong significant positive correlation (0.82-0.86) was noted between the size-related parameters (SSDE_BMI [non-specific]_, SSDE_SUM Dimension_, SSDE_AP Dimension_, SSDE_Effective diameter_ and SSDE_LAT Dimension_). The clinical DRL values based on the DLP and CTDI_vol_ of this study were compared with the DRL values published by Habib Geryes et al.^19^ as seen in Table 3.

**TABLE 2:** Diagnostic reference levels based on the median and 75th percentile, for all dose metrics. The mean image quality index (Hounsfield unit) and range (minimum–maximum) for non-specific body mass index and all body mass index classifications are the same for all dose metrics. Only body mass index classifications that had 20 or more patients per size category are included in the table.

Dose metrics	Non-specific BMI (*N* = 201)					Spearman correlation coefficient‡ (*N* = 201)	Normal weight (*N* = 65)					Overweight (*N* = 57)					Obese (*N* = 63)					Overweight vs Obese	Dose for two scan phases (*N* = 181)		Dose for three scan phases (*N* = 20)		Two scan phases vs three scan phases§
	Dose		Image quality index (HU)				Dose		Image quality index (HU)			Dose		Image quality index (HU)			Dose		Image quality index (HU)								
	Median	75th	Mean	Min	Max		Median	75th	Mean	Min	Max	Median	75th	Mean	Min	Max	Median	75th	Mean	Min	Max	*p*	Median	75th	Median	75th	*p*
SSDE_BMI (non-specific)_ (mGy)	12.6	14.5	15.99	8.71	39.84	-	10.7	12.5	15.91	11.25	39.84	14.7	15.7	5.32	11.17	23.90	13.3	14.1	16.67	11.75	23.34	0.0015	12.5	14.3	14.2	15.8	0.0013
SSDE_AP Dimension_ (mGy)	14.1	15.5	-	-	-	0.82	10.7	12.6	-	-	-	15.0	16.1	-	-	-	15.5	15.9	-	-	-	0.1094	14.1	15.5	14.5	16.0	-
SSDE_LAT Dimension_ (mGy)	14.1	16.4	-	-	-	0.86	12.0	13.7	-	-	-	15.9	17.4	-	-	-	15.4	17.3	-	-	-	0.7621	13.8	16.4	15.5	16.6	-
SSDE_SUM Dimension_ (mGy)	14.4	16.2	-	-	-	0.86	11.3	12.8	-	-	-	15.9	16.5	-	-	-	15.5	16.6	-	-	-	0.5036	14.4	16.1	15.5	16.5	-
SSDE_Effective Diameter_ (mGy)	14.2	15.9	-	-	-	0.86	11.1	12.7	-	-	-	15.6	16.4	-	-	-	15.5	16.4	-	-	-	0.5172	14.1	15.8	14.8	16.4	-
CTDI_vol_ (mGy)	11.6	13.6	-	-	-	-	7.5	9.6	-	-	-	12.2	13.1	-	-	-	13.8	13.9	-	-	-	-	11.4	13.5	12.7	13.8	0.0321
DLP† (mGy.cm)	1133.0	1280.0	-	-	-	-	749.0	898.9	-	-	-	1170.0	1270.0	-	-	-	1286.0	1351.0	-	-	-	-	1044.0	1249.1	1668.0	1817.3	<0.0001

Note: Normal weight (18.5 kg/m^2^ – 24.9 kg/m^2^); overweight (25 kg/m^2^ – 29.9 kg/m^2^); obese (≥ 30 kg/m^2^).

AP, anterior-posterior; BMI, body mass index; CTDI_vol_, volumetric CT dose index; DLP, dose-length product; HU, Hounsfield unit; Min, minimum, Max, maximum; LAT, lateral; mGy, milligray; mGy.cm, milligray centimetre; SSDE, size-specific dose estimates; SUM, AP + LAT dimension; 75th, 75th percentile.

† , calculated as the product of CTDI_vol_ and the scan length;

‡ , SSDEBMI_(non-specific)_ with SSDE_AP Dimension_ SSDE_LAT Dimension_ SSDE_SUM Dimension_ SSDE_Effective Diameter_;

§ , Median values.

**TABLE 3:** Comparison of diagnostic reference level values of this study with diagnostic reference level values published by Habib Geryes et al.^19^ based on the median and the 75th percentile for patient size categories which had 20 or more patients per category.

No. patients and dose metrics	Category	BMI category										No. scan phases			
		Non-specific BMI		Underweight		Normal weight		Overweight		Obese		Two		Three	
		This study	Habib Geryes et al.	This study†	Habib Geryes et al.	This study	Habib Geryes et al.	This study	Habib Geryes et al.	This study	Habib Geryes et al.	This study	Habib Geryes et al.	This study	Habib Geryes et al.
No. patients		*n* = 201	*n* = 467	*n* = 16	*n* = 24	*n* = 65	*n* = 193	*n* = 57	*n* = 98	*n* = 63	*n* = 41	*n* = 181	*n* = 136	*n* = 20	*n* = 44
CTDI_vol_ (mGy)	Median	11.6	-	-	-	7.5	-	12.2	-	13.8	-	11.4	-	12.7	-
CTDI_vol_ (mGy)	75th	13.6	-	-	-	9.4	-	13.1	-	13.9	-	13.5	-	13.8	-
DLP (mGy.cm)‡	Median	1133	763	-	436	749	666	1170	874	1286	1363	1044	890	1668	1273
DLP (mGy.cm)‡	75th	1280	1141	-	487	898.9	874	1270	1388	1351	1670	1249.1	1350	1817.3	1557

*Source*: Habib Geryes B, Hornbeck A, Jarrige V, Pierrat N, Ducou Le Pointe H, Dreuil S. Patient-dose evaluation in computed tomography: A French national study based on clinical indications. Phys Med. 2019;61:18–27. https://doi.org/10.1016/j.ejmp.2019.04.004

Note: Underweight (< 18.5 kg/m^2^); normal weight (18.5–24.9 kg/m^2^); overweight (25–29.9 kg/m^2^); obese (≥ 30 kg/m^2^).

BMI, body mass index; CTDI_vol_, volumetric CT dose index; DLP, dose-length product; mGy, milligray; mGy.cm, milligray centimetre; 75th, 75th percentile.

† , DRL values were not established for the BMI classification ‘underweight’ in this study because there were less than 20 patients;

‡ , calculated as the product of the CTDI_vol_ and the scan length.

The noise index (NI) at the medical oncology department was kept constant for all the CT CAP examinations, as displayed in Table 1. The liver displayed in the axial portal venous phase of the CT examination was used to measure the image quality objectively in this study for all patients and the different BMI classifications, as seen in Table 2.

## Discussion

The CT unit is not equipped with the latest available technology, such as adaptive statistical iterative reconstruction (ASIR), to promote further radiation dose reduction to the patient, which is a common scenario in resource-restricted public healthcare facilities in South Africa. Financial constraints in government institutions could be one of the reasons why the latest technology was not used at the participating department. However, the ATCM was activated at the medical oncology department to reduce the dose by adjusting the exposure parameters according to patient sizes, while maintaining the image quality of the CT images.^33,34,35^ Objective image quality assessment of the CT images was done using MathWorks^®^ imtool3D_td in MATLAB (https://www.mathworks.com/matlabcentral/fileexchange/74761-imtool3d_td), which was satisfactory for all the patients included in the study.

In prior studies,^12,34^ the image quality was measured over various regions of the body for CT CAP and abdomen-pelvis, such as the aorta, liver and spleen. However, in a study by Moghadam et al.^11^ investigating CT chest examination, the image quality was measured over the aorta, while for the CT abdominal examination, the image quality was measured over the liver. Therefore, for this study, the image quality was only measured over the liver. Even though the image quality was assessed with only one anatomical ROI, the quality of the CT images was satisfactory for all the patients included in the study. The mean standard deviation image quality index for this study was 15.99 HU (range 8.71 HU – 39.84 HU). The noise index was fairly constant in this study, which was similar to previously published findings.^36,37^ Furthermore, a radiology report was provided for all the examinations included in the study at the participating hospital, which also indicated that the images were of acceptable diagnostic value.

The axial portal venous phase was used to assess the image quality in this study, as described by Ahmad et al.,^37^ although these authors used a different slice thickness (2.5 mm – 3 mm) compared to this study which normalised the slice thickness to 5 mm. The image quality index for this study was higher (15.99 HU) than that published by Ahmad et al.^37^ (10.25 HU – 11.75 HU), who also reported on CT DRL values for oncology. It was expected that differences would be observed in the image quality index because of different CT units, reconstruction algorithms and different noise levels set for CT CAP imaging.^37^ Furthermore, subjective image quality was assessed by a radiologist who compiled the radiological reports for the CT images that were included in this study, with no negative comments on image quality.

Clinical DRL values based on the median value of the dose metrics for the patients included in the study and the 75th percentile for those dose metrics to give an indication of the range of the dose metric values could only be calculated for the most common cancers recorded in this study because 20 or more patients are usually considered to be sufficient to establish DRL values, as indicated by the ICRP.^14^ Therefore, cancers such as penile, renal cell carcinoma and metastatic pancreatic cancer were not included in the specific cancer results. Consequently, clinical DRL values for specific cancers were determined for only 144 patients.

Although the same anatomical region was scanned for the same main clinical indication (cancer), the dose differed among the various cancers. There were no significant differences (*p* = 0.12) between the median values of the SSDE_BMI (non-specific)_ for the specific types of cancer, which was expected, because the same clinical protocol was used, also described by Pema et al.^3^ Despite not finding significant differences of the radiation dose (SSDE_BMI [non-specific]_) among the specific cancers, calculating clinical DRL values will enable radiology departments to easily identify areas to review and/or adjust their respective protocols, while ensuring that optimal image quality is maintained to provide a diagnosis.

Clinical LDRL values were also calculated for non-specific cancers (all cancers) and the number of scan phases (201 patients) (Table 2). Underweight patients were excluded because there were less than 20 patients in that category. Furthermore, the image quality index based on these two variables is also highlighted in Table 2. All the patients were injected with contrast medium intravenously. The majority of patients received two scan phases (arterial and portal venous) for CT CAP examinations, while 20 (10.0%) patients received three scan phases (arterial, portal venous and delay). An increase in the number of scan phases resulted in a dose increase for all dose metrics, which was also evident in previous studies.^23,24^

A significant difference between the median values for SSDE_BMI (non-specific)_ (*p* = 0.0013), DLP (*p* < 0.0001) and CTDI_vol_ (*p* = 0.0321) was observed for the number of scan phases (two or three). Radiologists decide on the number of scan phases (protocol) a patient should receive based on the pathology and clinical history indicated on the X-ray request form. It is important that the protocol applied for cancer diagnosis examinations is justified and that the information provided by the scan phases contributes to image diagnosis.^24^

The radiation dose increased for all the dose metrics when the BMI increased from normal weight to obese (Table 2). However, overweight patients received a higher dose than obese patients for the specific size-related SSDE dose metrics SSDE_BMI (non-specific)_, SSDE_LAT Dimension_, SSDE_SUM Dimension_ and SSDE_Effective diameter_, as displayed in Table 2. A possible reason for this could perhaps be attributed to the different patient habitus^38^ and weight distributions because the abdomen consists of various structures that need to be penetrated during scanning when ATCM is used. When a patient is not positioned in the centre of the gantry during CT imaging, the functionality of the ATCM can be influenced^39^ and could be a possible reason for overweight patients having a higher dose compared to the obese patients in this study. No significant differences were observed between the overweight and obese patients for the SSDE_AP Dimension_ (*p* = 0.1094), SSDE_LA__T Dimension_ (*p* = 0.7621), SSDE_SUM Dimension_ (*p* = 0.5036) and SSDE_Effective diameter_ (*p* = 0.5172). However, a significant difference was noted between the overweight and obese patients for SSDE_BMI_ (*p* = 0.0015). A possible reason why a significant difference occurred between overweight and obese patients for SSDE_BMI_ could be because there are little to no differences among researchers in measuring the BMI, whereas researchers measure the AP and LAT dimensions differently to calculate the SSDE.^31^

The comparison of the DRL values between this study and that of Habib Geryes et al.^19^ was based on the distribution of median values per facility according to ICRP recommendations.^1^ The data reported by Habib Geryes et al.^19^ were collected over 3 years (2015–2017) from 88 CT units across France, some of which had been installed prior to 2007, the year that the CT unit in this study had been installed. Their pathology categories each included 15–30 consecutive examinations and included CAP tumours that were similar to those reported in this study. Habib Geryes et al.^19^ also compiled DRL values for the different BMI classifications and the number of scan phases, and therefore, their results could be meaningfully compared with this study’s findings. In relation to the number of scan phases, Habib Geryes et al.^19^ established clinical DRL values for one, two, three and four scan phases for CAP tumours. For comparison purposes, only the DRL values for two and three scan phases as published by Habib Geryes et al.,^19^ were recorded in Table 3. The DRL values for CAP cancer were higher in this study compared to those reported by Habib Geryes et al.,^19^ because of a lower DLP. However, obese patients in this study had a lower DRL value (DLP) compared to patients in the Habib Geryes et al^19^ study.

The overweight patients with breast cancer had a DLP of 1077.8 mGy.cm, while the DLP was 1263.0 mGy.cm for obese patients based on the median. Clear differences for the DLP were noted among the BMI classifications for breast cancer. A significant difference (*p* < 0.0001) was also observed between the median values for SSDE_BMI_ for the different BMI classifications, which emphasises the importance of calculating clinical DRL values for specific BMI groups as well.

The DLP differed among the specific cancers, which might be attributed to the longer scan lengths that were obtained. Among the three cancer groups, GIT organ-related cancer had a longer mean scan length of 394 mm compared to breast cancer and cancer of the reproductive system, which had a mean scan length of 379 mm and 388 mm, respectively. A feasible explanation could be that a longer scan length was used for patients with oesophageal, stomach, colon and/or rectosigmoid, anal, rectal and caecum cancer not to miss any possible metastases, and therefore, the DLP increased, which was also evident in previous studies.^5,7^

A strong positive correlation (0.82–0.86) was noted between the size-related parameters; therefore, it can be concluded that any size-related parameter can be used to determine the SSDE for cancer patients. However, using the BMI instead of the AP and LAT dimensions to calculate the SSDE will result in minimal measurement inconsistency.^31^ Moreover, a significant correlation (*p* < 0.0001) was also observed between the size-related parameters. A significant difference (*p* < 0.0001) between the CTDI_vol_, DLP and the SSDE for all the BMI classifications was noted, indicating a significant effect of these quantities on the DRL. Deciding on which DRL quantity to use in CT imaging to develop DRL values is important. Research has shown that it is better to use the SSDE to calculate patient doses because the DRL quantity is based on the size of the patient.^40^ Furthermore, there is a significant strong positive correlation (0.64–0.86) between the DLP, CTDI_vol_ and the SSDE for all the BMI classifications. As the BMI increases, the dose the patient receives also increases.

A convenience sampling technique was used to select the participating medical oncology department because the department was in close proximity to the researcher. The patients who met the inclusion criteria at the participating medical oncology department were also selected using the convenience sampling technique, because these patients were already booked for CT CAP examinations, their clinical indication was cancer-related and the patients were easily accessible. However, a good representation of the underweight patients could not be achieved using the convenience sampling technique. As a result, the main limitation noted for the study was that the clinical DRL values could not be developed for underweight patients because there were less than 20 patients in this BMI classification. Another limitation was that only one medical oncology department was included in the study to evaluate the patient doses and image quality metrics. Additionally, an image quality scoring system was not utilised to assess subjective image quality. Because the radiologist reported on the CT images without commenting negatively about the image quality, the authors presumed that the image quality was of diagnostic value. Furthermore, the patient doses and image quality metrics calculated only focussed on CT CAP examinations for adult patients. Moreover, the image quality was only determined on the liver in this study and not on the other internal organs such as the spleen and intestines.

Future studies are necessary to determine DRL values based on the clinical indication for underweight patients, and to investigate a larger variety of different types of cancer, evaluating them in terms of BMI classifications and the same number of scan phases. In addition, an image quality scoring system should be utilised to assess subjective image quality when clinical DRL values are established. Furthermore, image quality metrics and clinical DRL values should be assessed for paediatric CT examinations in future studies.

## Conclusion

At the time of the study, no baseline DRL values were available at this particular medical oncology department. For future diagnostic CT examinations, the results of this study could be applied as a starting point. Dose differences occurred among the specific cancers and the different BMI classifications. Hence, it is essential to calculate DRL values based on patients’ sizes. Furthermore, significant differences were also noted among the number of scan phases, which clearly indicated that the number of scan phases influences the dose. The quality of these images was diagnostically and clinically adequate for drawing a conclusion on the patients’ cancer status.
